# Intratumoral Switch of Molecular Phenotype and Overall Survival in Muscle Invasive Bladder Cancer

**DOI:** 10.3390/cancers14133256

**Published:** 2022-07-02

**Authors:** Camilla De Carlo, Marina Valeri, Noemi Rudini, Paolo Andrea Zucali, Miriam Cieri, Grazia Maria Elefante, Federica D’antonio, Rodolfo Hurle, Laura Giordano, Alessandra Bressan, Massimo Lazzeri, Matteo Perrino, Giorgio Guazzoni, Luigi Maria Terracciano, Piergiuseppe Colombo

**Affiliations:** 1 Department of Pathology, IRCCS Humanitas Research Hospital, Via Manzoni 56, Rozzano, 20089 Milan, Italy; camilla.decarlo@humanitas.it (C.D.C.); marina.valeri@humanitas.it (M.V.); noemi.rudini@humanitas.it (N.R.); miriam.cieri@humanitas.it (M.C.); grazia.elefante@humanitas.it (G.M.E.); alessandra.bressan@humanitas.it (A.B.); luigi.terracciano@hunimed.eu (L.M.T.); 2 Department of Biomedical Sciences, Humanitas University, Via Rita Levi Montalcini 4, Pieve Emanuele, 20090 Milan, Italy; paolo.zucali@hunimed.eu (P.A.Z.); federica.dantonio@humanitas.it (F.D.); giorgio.guazzoni@hunimed.eu (G.G.); 3 Department of Oncology, IRCCS Humanitas Research Hospital, Via Manzoni 56, Rozzano, 20089 Milan, Italy; matteo.perrino@humanitas.it; 4 Department of Urology, IRCCS Humanitas Research Hospital, Via Manzoni 56, Rozzano, 20089 Milan, Italy; rodolfo.hurle@humanitas.it (R.H.); massimo.lazzeri@humanitas.it (M.L.); 5 Biostatistic Unit, IRCCS Humanitas Research Hospital, Via Manzoni 56, Rozzano, 20089 Milan, Italy; laura.giordano@humanitas.it

**Keywords:** bladder cancer, molecular phenotype, luminal, basal, neu-like, phenotypical transition, *CD44*, *CK5/6*, *CK20*, *pPARγ*, OS, PFS

## Abstract

**Simple Summary:**

The Cancer Genome Atlas (TCGA) and more recent genome profiling recently revealed major intrinsic molecular subtypes in urothelial carcinoma (UC). Here we propose a fast and standardized immunophenotypical classification score (Piescore) that may discriminate between luminal, basal, or neu-like UC as a surrogate of molecular profile, and we describe, for the first time, an intratumoral phenotypical switch in tissue protein expression, from non-muscle to muscle-invasive progression. Our data show that a change from a luminal to a neu-like phenotype could worsen overall survival compared with a transition to a basal phenotype.

**Abstract:**

In recent years, immunohistochemical protein expression was studied as a surrogate to the molecular classification of bladder cancer, although no tissue biomarkers are available for clinical use to predict survival or the response to neoadjuvant chemotherapy (CT) in UC, as the literature produced conflicting results. This retrospective study included TURB specimens harboring foci of HG pT2 muscle-invasive bladder carcinoma (MIBC) from 251 patients who subsequently underwent radical cystectomy. We performed immunohistochemical analysis on tumor samples, for relevant gene-expression-based markers for basal type (*CD44*, *CK5/6*) and luminal type (*CK20* and *pPARγ*). Piescore, investigated in both non-muscle-invasive (NMI) and muscle-invasive (MI) components of the tumor, divided basal and luminal UC-types when at least three of the four markers were consistent with a specific phenotype, mixed types if one/two luminal and basal markers were present simultaneously, and neu-like types when all four markers investigated were negative. Eighteen selected cases were also investigated with RT-PCR to validate, and to increase the specificity of, the immunohistochemical results. We observe an immunophenotypical difference in the NMI and MI components in 96/251 UC patients (38.25%): half of tumors (44/96 cases) have a transition to basal, 36.46% (35/96 cases) to neu-like, 12.5% (12/96 cases) to mixed, and 5.2% (5/96 cases) to luminal phenotypes. Mixed tumors in the NMI component are more likely to change phenotype than other groups, particularly compared with basal tumors, which demonstrate greater stability (only 8/96 cases, *p* < 0.00001). The transition of luminal tumors to basal display a better OS compared with the transition toward neu-like tumors (*p* = 0.027). Overall, the phenotypical switch does not affect lymphovascular invasion, pT, DFS, or OS compared with non-switched cases. In the MI component, the presence of *CD44* expression, irrespective of score-related phenotype, shows a protective effect in papillary-type UC (OS *p* = 0.008, HR 0.453, PFS *p* = 0.07, HR 0.599), and in UC naïve for CT (*p* = 0.0479). Piescore immunophenotyping reveals an intratumoral phenotypical transition between the NMI and MI components of the same tumor. The molecular change is a common event in the mixed and luminal categories, but not in basal tumors, which show better phenotypical stability. This phenomenon could partially explain the sensitivity of a subset of luminal UC to chemotherapy: good responders could be “non-real” luminal UC, which acquire nasal markers, such as *CD44*.

## 1. Introduction

Urothelial bladder carcinoma (UC) represents the fourth most frequent cancer in highly industrialized countries [1,2,3,4]. More than 50% of patients experience metastasis and death, despite systemic and surgical therapies [5]. Treatment for MIBC patients consists of neoadjuvant therapy followed by radical cystectomy, pelvic lymph node dissection, and urinary diversion [6,7]. Although radical cystectomy (RC) remains the mandatory treatment for MIBC, this approach maintains significant post-surgical implications, and strongly influences the patient’s life [8]. Despite recent improvements, the prognosis of patients with advanced or metastatic UC remains poor, with a median overall survival (OS) of approximately 15 months from diagnosis [9]. The therapeutic approach to UC has been dominated by cisplatin-based chemotherapy (CT) for many years. Now, the management of UC treatment is shifting towards a more personalized approach. For this reason, new agents in advanced or metastatic UC were validated in recent years, including immune checkpoint inhibitors (ICI), tyrosine kinase inhibitors (TKI) targeting fibroblast growth factor receptors (FGFR), and antibody–drug conjugates (ADC) directed against Nectin-4 [10]. Although many clinico-pathological risk parameters are considered to predict the outcome of the disease, and to provide information for clinical decision-making, there are some limitations, due to the frequently unpredictable behavior of this tumor, often in the early stages of the disease.

The discovery of new gene markers in bladder cancer provided more precise and reliable information on clinical behavior, response to therapy, and patient risk-profile [11,12]. In the last decade, a TCGA collaborative project uncovered major intrinsic molecular subtypes in UC: basal, luminal, and neural-like MIBC, which harbor different prognostic and predictive values [13,14,15]. Later, further classifications, based on this gene expression profile approach, were proposed, either in non-muscle-invasive (NMIBC) or in MIBC, often using non-overlapping datasets, and different methods. In 2019, a consensus classification converged on six different relevant molecular classes: luminal papillary, luminal not specified, luminal unstable, stroma-rich, basal/squamous, and neuroendocrine-like, with basal/squamous and luminal papillary being the largest categories (35% and 24% of cases, respectively) [16]. In this paper, immunohistochemical staining was able to discriminate broad categories of luminal and basal types acting as surrogates; nevertheless, an agreement on which markers have to be used to discriminate the different categories is lacking.

Furthermore, Rebola et al., using an immunohistochemical two-cytokeratin panel (*CK20*, *CK5/6*) in pT1 UC, also support a dichotomic luminal/basal-based classification, which can be a fast and low-cost predictor of disease behavior that is easy to transfer into clinical practice [14].

Among the investigated biomarkers, *CD44* and *pPARγ* prove useful in distinguishing the different subtypes [17,18]. Basal urothelial carcinoma shows a positive stain for *CD44* and *CK5/6,* and a negative stain for *CK20* and *pPARγ*, whereas luminal urothelial carcinoma shows an opposite pattern [19,20].

A standardized, inexpensive, and fast method to classify the different molecular subtypes in UC remains to be determined. In the present paper, we propose a new immunophenotypical classification score (Piescore) as a simple surrogate, able to stratify different MIBC between luminal vs. basal subtypes. Furthermore, we describe, for the first time in the literature, an intratumoral heterogeneity in luminal–basal phenotypical expression during the progression from the NMI to MI components; we discuss the possible correlation between the Piescore and clinic-pathological variables in a mono-institutional cohort of UC treated with TURB, and subsequent RC.

## 2. Materials and Methods

### 2.1. Case Selection

This retrospective monocentric study, approved by our Institutional Review Board and local ethical committee (No. Uronca-001 v1.0), included all patients with MIBC (pT2) who underwent TURB and RC at IRCCS Humanitas Clinical and Research Hospital between 2000 and 2018, with available follow-up data. Exclusion criteria was the presence of previous and/or concurrent malignancies (except tumors for which the patient was disease-free prior to bladder carcinoma diagnosis). 

### 2.2. Pathological and Immunohistochemical Evaluation

From the archives of the Pathology Department, Humanitas University, representative paraffin block from each tumor was cut to obtain 2u sections for immunostaining. The following primary monoclonal antibodies were used: *CD44* (clone SP37, Ventana Roche, Oro Valley, AZ, USA), *CK5/6* (clone D5/16B4, Ventana Roche), *CK20* (clone SP33, Ventana Roche), and *pPARγ* (clone SC7273, Santa Cruz). Immunohistochemistry was performed using a Ventana automated system (Ventana Roche).

Sections were counterstained with Mayer’s hematoxylin. Positive and negative controls were present in the slides. Two expert uropathologists (P.C.; G.M.E.) evaluated all cases, blind to clinical information, and reviewed discordant results to reach a consensus.

For each tumor, the presence of immunoreactivity for all four markers was analyzed in both the superficial (papillary or solid) and deep-muscle-invasive component, and the results recorded separately. Positivity for *CD44* was defined as immunoreactivity in tumor cells with a membrane pattern of staining; *CK5/6* and *CK20* were characterized by a cytoplasmic/membrane staining pattern; and *pPARγ* had a nuclear staining.

In order to make the immunophenotypical evaluation, we conceived an integrated scoring system, called Piescore, which was able to minimize the overestimation of staining positivity.

The Piescore system was based on the evaluation of the percentage of positive cells and staining intensity: a tumor was scored as ‘0’ if <10% of tumor cells stained positive, ‘1’ if 10% to 25% were positive, ‘2’ if 25% to 50% were positive, and ‘3’ if >50% of cells showed positivity. Staining intensity was scored as ‘0’ when no cells were stained, ‘1’ for weak staining, ‘2’ for moderate staining, and ‘3’ in the case of strong staining. The sum of the scores for positivity and intensity determined a final score (Piescore), ranging from 1 to 6; cases with Piescore of ≥4 were considered positive.

### 2.3. Identification of Luminal, Basal, Mixed, or Neu-Like Phenotype in pT2 Carcinoma

Positivity for *CD44* and/or *CK5/6* defined tumors with basal differentiation, while positivity for *CK20* and/or *pPARγ* defined luminal differentiation. Positivity for at least one of the two markers in both categories (luminal and basal) identified tumors with mixed differentiation. Furthermore, absence of all markers identified a neu (Null) phenotype (so called neuroendocrine-like). 

**Figure 1:**
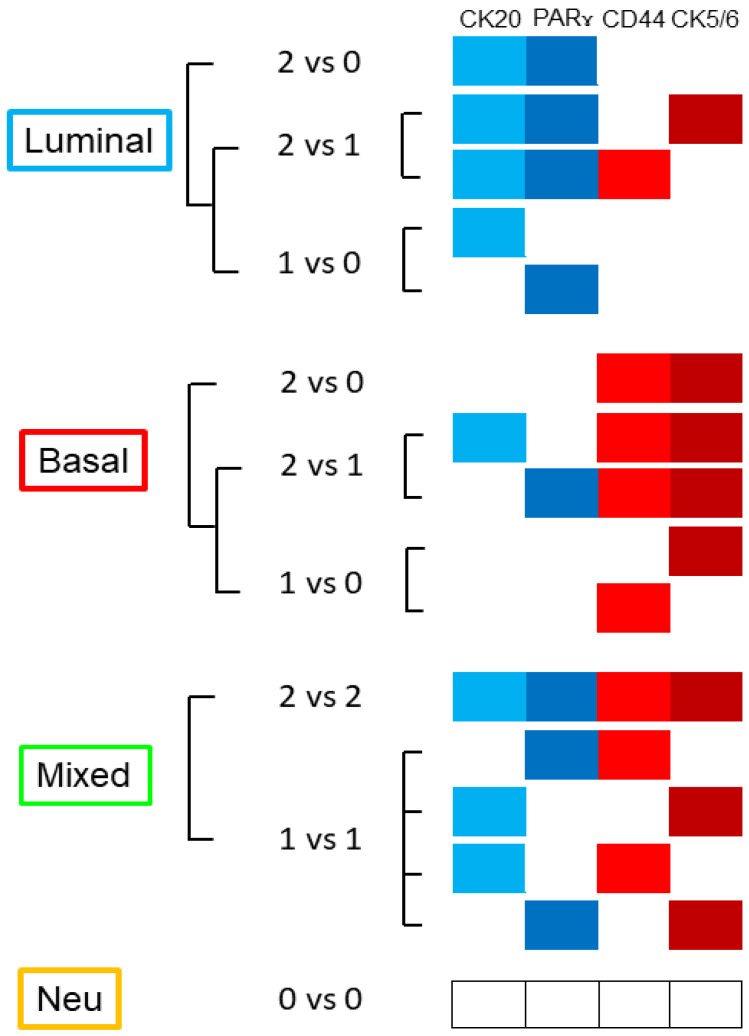
Basal phenotype is defined when at least 3 markers consistent with the basal phenotype are documented; luminal when at least 3 markers consistent with the luminal phenotype are noted; mixed when 1 luminal and 1 basal marker are expressed simultaneously, or 2 luminal and 2 basal markers are expressed simultaneously; and neu-like when 4 markers are negative.

The basal group was defined by positive expression of at least one basal marker (*CD44*+ and/or *CK5/6*+) and negativity of at least one luminal marker (*CK20* and *pPARγ*). The luminal group was defined by positivity for at least one luminal marker (*CK20* and/or *pPARγ*) and negativity of at least one basal marker (*CD44* and *CK5/6*); the mixed group was characterized by positivity for all markers, or positivity in one marker from both categories; the neu group had negative expression of all markers. For details on markers used and combinations of markers, see Figure 1.

### 2.4. Molecular Identification of CD44, CK5/6, CK20, and pPARγ through qRT-PCR Assay

From the analyzed cohort, we selected 18 MIBCs for validation purposes. Representative tumor areas were properly microdissected from each sample block. Gene expression of *pPARγ*, *CK5/6*, *CK20,* and *CD44* was tested by RT-PCR. Briefly, RNA was extracted from paraffin-embedded bladder cancer tissue, at least 60% tumour-rich, using the Maxwell RSC RNA FFPE Kit (Promega, Madison, WI, USA), according to the manufacturer’s instructions. Manual microdissection was performed to select regions of interest. A total of 50–450 ng of RNA was reverse-transcribed using SuperScript™ IV VILO™ Master Mix (Life technologies, Carlsbad, CA, USA), using random hexamers, according to the manufacturer’s instructions. A 2ul cDNA template was amplified with the Taqman Gene Expression Assay using TaqManFast Advance Mix (Applied Biosystems, Waltham, MA, USA), using QuantStudio 12K Flex Real Time PCR 96-well plate system. The mRNA expression of the following genes was tested using precast Taqman probes: *CK5* (HS 00934200_g1_FAM_MGB), *CK20* (HS00300643_m1_FAM_MGB), *pPARγ* (HS00234592_m1_FAM_MGB), and *CD44* (HS99999195_m1_FAM_MGB). The following protocol of qPCR was used: 50 °C 2 min (UNG incubation), 95 °C 2 min (polymerase activation), 40 cycles for 95 °C 3 s (denaturation), and 60 °C 30 s (annealing/extension). For any sample in duplicate, the expression level, normalized to the housekeeping gene encoding Gapdh, was determined by the comparative threshold cycle (Ct) method, as previously described [21]. Gene expression was calculated using control samples (non-tumoral-adjacent tissue). A relative quantification above 1 represents upregulation, and below 1 represents downregulation.

Statistical analysis was performed using Prism Software, with the D’agostino–Pearson test as a normalization test.

### 2.5. Statistical Analysis

Data were summarized as frequency and proportion, or as median and range. Chi-square test or Fisher’s exact test were used to compare categorical variables (expression of *CD44*, *CK5/6*, *CK20*, *pPARγ*; overall phenotypical categories; subtype of UC; and clinico-pathological variables). Progression-free and overall survival were calculated from surgery (cystectomy) to first progression, recurrence date, or last contact date, evaluated using the Kaplan–Meier method and differences between groups, then tested by log-rank test. The Cox proportional hazard model was used to estimate the hazard ratio (HR), with 95% confidence intervals (CIs). Statistical significance was set at 0.050.

All analyses were performed using SAS v.9.4 (SAS Institute Inc 2013. SAS/ACCESS® 9.4). 

## 3. Results

### 3.1. Clinical and Pathological Characteristics of Patients and Definition of the Different Molecular Groups

Among patients with a diagnosis of urothelial bladder carcinoma, from January 2000 to December 2018, a series of 251 consecutive patients with MIBC (pT2) at TURB followed by radical cystectomy are included in this study. Clinical features of patients are shown in Table 1.

The mean age at TUR is 68 years (range 49–89 y; median 69 y). The male to female ratio is 4.5:1. “Primary” tumors (MIBC at first diagnosis on TURB) are documented in 219 patients, while “secondary” tumors (initially pTa-pT1 carcinoma with subsequent progression to pT2) are present in 32 patients. Papillary vs. non papillary tumors are documented in 55.38% (139/251) and 44.62% (112/251) of patients, respectively. Vascular invasion (LVI) is present in 40.64% (102/251) of cases. Necrosis is detected in 38.25% (96/251) of cases. At cystectomy, the tumor stage is stratified as follows: pT0 (*n* = 24; 9.56%), pTis/Ta (*n* = 8; 3.19%), pT1 (*n* = 9; 3.58%), pT2 (*n* = 29; 11.55%), pT3a-b (*n* = 139; 55.38%), and pT4 (*n* = 42; 16.73%). At the time of cystectomy, 58 patients (23.11%) have lymph node metastasis, while 5 patients (2%) are pM1.

### 3.2. Immunohistochemical Expression of CD44, CK5/6, CK20, and pPARγ and Identification of the Different Categories

**Figure 2:**
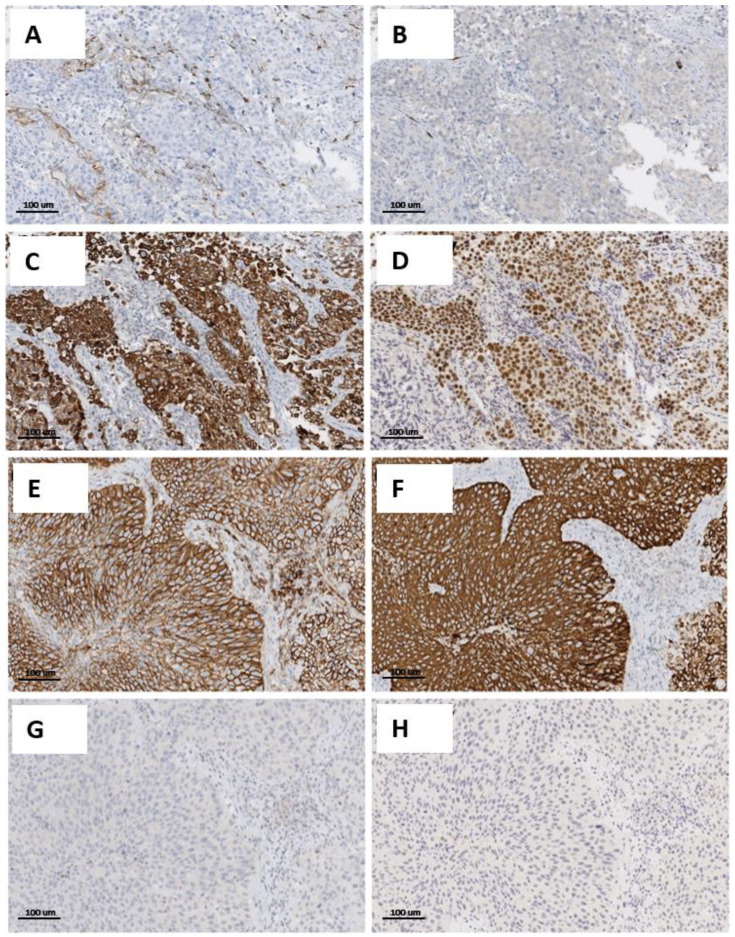
Different and opposite markers expression in luminal and basal tumors. (**A**–**D**): prototypical example of a luminal tumor. (**A**) Negative staining for CK5/6; (**B**) negative staining for CD44; (**C**) strong positivity for CK20; (**D**) nuclear positive staining for pPARγ. (**E**–**H**): prototypical example of a basal tumor. (**E**) Positive staining for CK5/6; (**F**) positive staining for CD44; (**G**) negative staining for CK20; (**H**) negative staining for pPARγ. IHC stain, 200×.

The pathological characteristics of patients, and corresponding tumors and phenotypes, are summarized in Table 1. Overall, in the NMI component, luminal, basal, mixed, and neu-like phenotypes are observed in 121 (48.21%), 71 (28.29%), 39 (15.54%), and 20 (7.97%) cases, respectively. The MI component results in basal in 107 pts (42.63%), while 73 pts (29.08%) are luminal type, 21 pts (8.37%) are mixed type, and 50 pts (19.92%) are neu type. Figure 2 illustrates two prototypical examples of immunohistochemical expression in luminal and basal tumors.

The different phenotypic categories do not show correlation with age, gender, smoking history, primary vs. secondary tumor, associated CIS, LVI, necrosis, or pT at cystectomy.

Table 2 shows different markers expression stratified for histological characteristics. Papillary and non-papillary morphology demonstrate an equal distribution among the different phenotypes, with papillary architecture documented in 36/73 luminal, 63/107 basal, 28/50 neu-like, and 12/21 mixed tumors.

In contrast with the classic UC group, where all the phenotypical category results are equally distributed, tumors with divergent differentiation are mostly in the basal group (49/71, 69.01%), as well as for all sarcomatoid tumors (five cases). Nested UC (five pts) is equally distributed, micropapillary UC are all luminal (two pts), while plasmacytoid cases (three pts) and seven of eight neuroendocrine UC cases have a neu phenotype (Table 2). Interestingly, while 7/50 (14%) neu-like cases have morphology and phenotype consistent with neuroendocrine carcinoma (synaptophysin+, chromogranin+, not shown), the majority of cases harbor the usual urothelial carcinoma differentiation (43/50, 86%). Table 3 shows the possible combinations resulting from expression of the four markers.

**Figure 3:**
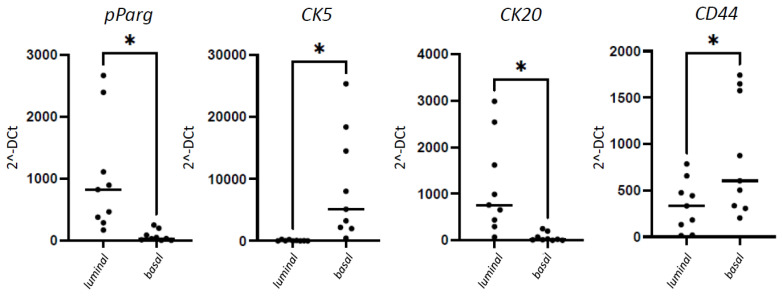
Gene expression of UC markers. Quantification of pPARγ, CK5, CK20, and CD44 gene expression in tissue specimens from 18 patients (black dots) with UC following RT-PCR analysis. mRNA expression for each gene is normalized to GAPDH. Total of 9 luminal vs. 9 basal UC subtypes: pPARγ * *p* = 0.014; CK20 * *p* = 0.0130; CK5 * *p* = 0.0167; CD44 * *p* = 0.0404.

Using the RT-PCR, selected cases (18 pts) undergoing amplification analysis confirm the immunohistochemical findings. The distinction of basal and luminal UC subtypes is evident, since the mean expression of *CK20* and *pPARγ* are significantly higher in luminal cases, as is *CK5* and *CD44* expression in the basal cases (see Figure 3).

### 3.3. Phenotypical Switch from Superficial to Muscle-Invasive Component of UC

A different phenotype for NMI compared to MI carcinoma is documented in 96/251 pts (38.2%) (Table 4). Specifically, phenotypical change is most common in the mixed (30/39) and luminal tumor groups (53/121), compared with the remaining categories (*p* < 0.00001). Interestingly, among 53 luminal switched patients, 44 cases (83%) have a complete transition (loss of both *CK20* and *pPARγ*): 18 cases become basal, while 26 cases switch to a neu-like phenotype. Overall, basal cases show the most stable phenotype, with only 8/71 patients harboring a transition (11.3%): four cases to neu, three cases to mixed, and one case to the luminal category.

Among 20 neu-like cases, 5 pts (25%) acquire a new phenotype: basal in 3 cases, and mixed and luminal in 1 patient each. 

**Figure 4:**
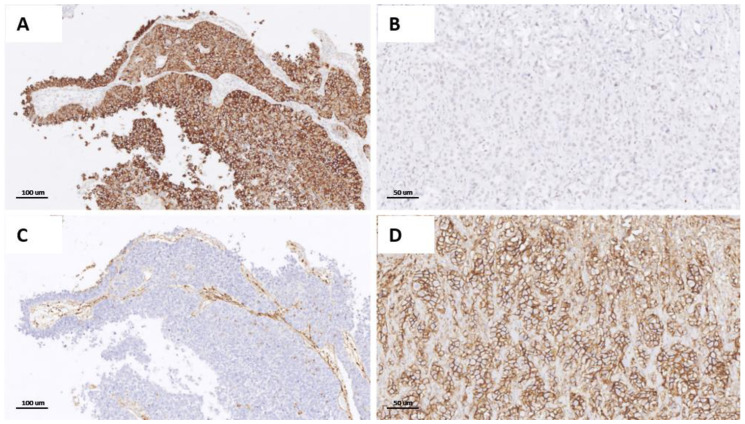
Immunophenotypical change from luminal to basal type in the superficial NMI component ((**A**,**C**), 200×), and in the MI compartment ((**B**,**D**), 400×) of the same tumor. (**A**) Superficial component with immunoreactivity for *CK20*; (**B**) MI component in same case with *CK20*-negative stain; (**C**) superficial NMI compartment with *CD44*-negative stain; (**D**) MI compartment with *CD44*-positive stain.

Papillary UC changes phenotype more frequently compared with non-papillary UC: 64/139 (46%) vs. 32/112 (28.57%) cases, respectively (*p* = 0.0046). No statistical differences between classic UC and variants are observed. Figure 4 illustrates an example of the transition from a luminal to basal phenotype in a papillary UC.

### 3.4. Histopathological Parameters and Outcome

**Figure 5:**
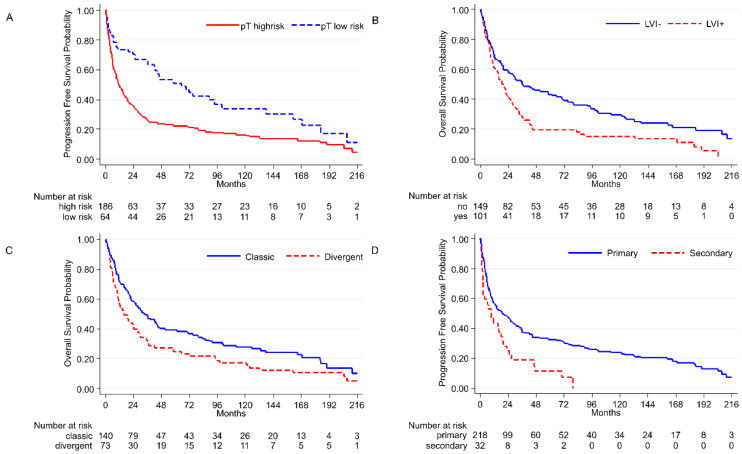
Most representative histopathological results: (**A**) High-risk stage (pT3-4 N0/N1) vs. low-risk stage (pT0-2 N0), PFS (*p* < 0.0001); (**B**) lymphovascular invasion, OS (*p* = 0.0006); (**C**) classic morphology vs. divergent morphology, OS (*p* = 0.0127); (**D**) primary vs. secondary tumors, PFS (*p* = 0.001).

Pathological parameters and outcome are summarized in Figure 5. The stratification of tumor staging in low risk (pT0-pT2N0) vs. high risk (pT3-4N0 or N1) shows a statistically significant difference in OS and PFS after RC (OS: *p* = 0.0005; PFS: *p* < 0.0001), with a worse outcome for the high-risk group (Figure 5A). This difference in PFS is also observed among cases naive to chemotherapy (*p* = 0.01). The presence of necrosis (OS and PFS: *p* = 0.02) and LVI (OS: *p* = 0.0006; PFS: *p* = 0.0008) provides a worse outcome (Figure 5B). Divergent differentiation also affects survival in OS (*p* = 0.0127), but not in PFS (Figure 5C). A statistically significant difference between primary and secondary tumors is also observed, with the former having a better outcome (OS: *p* = 0.002; PFS: *p* = 0.001) (Figure 5D).

### 3.5. Intratumoral Molecular Switch, Phenotypes, and Outcome

**Figure 6:**
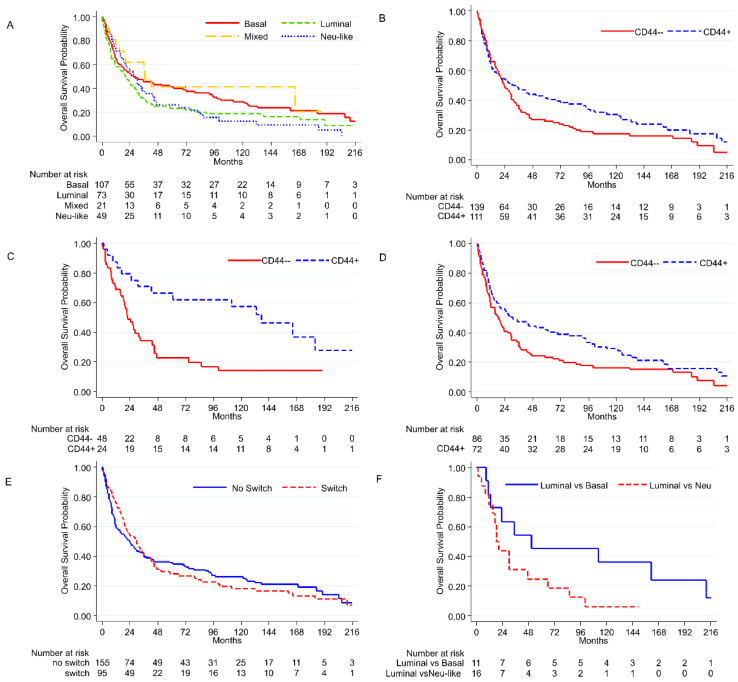
Distribution of mixed, basal, luminal, and neu-like phenotypes, OS (*p* = 0.09) (**A**); *CD44* expression in all tumors, OS (*p* = 0.079) (**B**); *CD44* expression in papillary UC, OS (*p* = 0.008) (**C**); *CD44* expression in CT naïve tumors, OS (*p* = 0.04) (**D**); switched vs. non-switched tumors, OS (*p* = 0.96) (**E**); switch towards basal patients vs. switch towards neu-like type patients, OS (*p* = 0.027) (**F**).

We do not find clear correlations between the four different molecular phenotypes and survival. Only a decreasing protective trend in PFS is noted when a mixed, basal, luminal, or neu-like group is identified (Figure 6A). The expression of *CD44* does not show a correlation with OS (*p* = 0.079, Figure 6B), but has a protective effect when considering only the papillary UC subtype (*p* = 0.008, HR 0.463), and in UC pts naïve to CT (*p* = 0.04, HR 0.61) (Figure 6C,D). Although less evident, and not statistically significant, *CK5/6* also shows a protective trend in papillary UC (*p* = 0.186, HR 0.625).

When comparing switching and stable (not-switching) cases for phenotypical transition, differences in OS and PFS are not observed (Figure 6E). A worse OS is detected for luminal tumors switching to neu-like (lum–neu), compared with luminal cases switching to basal (lum–bas) (OS: *p* = 0.027; HR 1,83) (Figure 6F). Conversely, no significant differences in OS and PFS are noticed between lum–bas and those retaining the luminal phenotype (lum–lum) (*p* = 0.109; HR 0.62). These data are also true for PFS (*p* = 0.10).

## 4. Discussion

The field of molecular subtyping is constantly evolving in bladder cancer but, given the high tumor response heterogeneity, many studies are ongoing to identify which patients will progress, or will be responsive to CT or immune therapy. The heterogeneity in response rate in bladder cancer is also reflected in its molecular profile, including the recently identified luminal/basal phenotypes. TCGA stratified five potential intrinsic subgroups (luminal papillary, luminal infiltrated, luminal, basal/squamous, and neuronal), with the former three types being luminal and the latter two basal cell differentiation. Choi et al., apart from genomic analysis, also divide luminal and basal tumors with immunohistochemistry, but currently no specific biomarker is available to accurately stratify these categories. Although a subset of biomarkers reached a consensus in the definition of basal tumors, and defining patients with such phenotypes responsive to cisplatin-based NAC, the use of this marker shows some limitations, and does not obtain clear, fast, and reproducible indications that can be replicated in daily practice [15].

In the present study, we carried out an extensive retrospective analysis to define major molecular subtypes in MIBC, with the belief that such subtypes may be objectively assessed, biologically relevant, and function as a complement to the current therapeutic approach. Among a consecutive series of pT2 urothelial carcinoma patients, our system identified four molecular subtypes using *CD44*, *CK5/6*, *CK20,* and *pPARγ*. The system is simple, fast, low-cost, and, as a surrogate, able to discriminate between luminal, basal, neu-like, or mixed UC. In our cohort, the basal phenotype is the most frequent group (107 cases, 42,6%), followed by luminal (73 cases, 29.08%), neu-like (50 cases, 19.9%), and mixed type (21 cases, 8.37%). In MIBC, the basal phenotype is recognized as a marker of a worse prognosis in OS by some authors, and is associated with upstaging after RC [16,22]. In our cohort, we fail to find a clear correlation between the basal phenotype and a worse prognosis (PFS *p* = 0.08; OS *p* = 0.09), as other authors have, so the real significance of this type of subclassification in bladder cancer remains to be understood [23]. 

Furthermore, a subset of our cases reveal a mixed phenotype, a phenomenon that supports the hypothesis that UC is a heterogeneous genomic disease that could harbor multiple clonal differentiations within the same tumor [24]. Although these patients represent only a small group, no prognostic correlations in OS and PFS are identified. 

In 2019, Batista da Costa et al. characterized a small group of bladder carcinomas called “neuroendocrine-like”, based on the absence of a luminal/basal molecular phenotype [25]. In the current study, we also note a group of patients with silent expression in all markers (50 pts), that we define as “Neu-like”. Interestingly, only seven patients have morphology of neuroendocrine carcinoma, while most cases (43 pts) have typical histological features of urothelial carcinoma. These results are unexpected, and are under further investigation. 

In line with data of the literature, all five sarcomatoid UC fit into the basal category, while all five nested UC are of the luminal type [26]. We also notice that divergent tumors are mostly in the basal group (49/71 cases, 69.01%), compared with the luminal tumors (11/71 of cases, 15.49%), as observed by other authors [26,27].

When considering the expression of the four markers analyzed individually, we observe a correlation between *CD44* and OS. This protective effect is most consistent in papillary tumors and in (all) UC naïve from CT, where it is plausible that tumors follow their natural history (Figure 5). Based on these results, our hypothesis is that when *CD44*, and, to a lesser extent, also *CK5/6*, is expressed by tumor cells in pT2 UC, it has an intrinsic power to stratify patients with the best outcome, regardless of luminal vs. basal subdivision, obtained with our score analysis (some of our luminal cases also express *CD44*; see Table 3). On the contrary, we identify no correlations for *CK20* and *pPARγ*.

Even if *CD44* expression predicts poor survival in many types of human cancers, demonstrated by some authors, the literature for urothelial carcinoma reveals conflicting results [17]. Desai et al., for instance, note that *CD44* positivity is a “protective” factor, with a loss of *CD44* increasing grade and stage in pTa and pT1 urothelial carcinoma [28]. These contrasting conclusions can be explained by the presence of several polymorphisms in *CD44*, which may or may not have a protective effect on the development of bladder cancer [28,29].

The molecular evaluation in our cohort shows an interesting phenotypical switch between the NMI component and MI component of the same tumor. To the best of our knowledge, this peculiar finding is not described in the literature. This intratumoral discordant expression is particularly evident in mixed tumors (30/39 cases), and appears to be related to the intrinsic molecular properties of this group.

The phenotypical heterogeneity is also well documented in NMI luminal tumors with 42.4% (53/121) of cases switching to other phenotypes (lum–bas, lum–mix), and with half of cases completely losing the expression of luminal markers (26/53, lum–neu). The complete loss of markers expression (neu-like) is significantly correlated with worse prognosis compared with acquiring a basal phenotype (OS: *p* = 0.027; HR 1.83) (Figure 6F).

NMI basal tumors, conversely, show a more stable phenotype in the MI component (only 8/71 cases have a transition), implying that tumors with a basal phenotype express a basal differentiation, even in the MI component. Phenotypical switch is observed in all subtypes of UC, but predominantly in papillary UCs (67% cases), which mostly results in a more unstable luminal or mixed phenotype (Table 2).

In the paper by Barth et al., studying the progression of a series of CIS to invasive carcinoma, the authors note a similar phenomenon, with a significant loss of luminal marker expression in the course of progression, and an increase in basal marker expression in the invasive compartment [30]. Data from TCGA partially supports these observations, in which a strong CIS signature gene expression is found mainly in the basal subgroup of MIBC, suggesting that basal tumors evolve from CIS. 

In the paper by Lee et al., the authors analyze a subset of urothelial organoids, and notice a phenotypical transition (luminal to basal) in most cases, starting from cell lines originated by the parental tumor toward the organoid, with a reversible phenomenon (basal to luminal) when organoids are inoculated in xenografts [31]. As suggested by the authors, the transition could be explained by cellular plasticity that originates from epigenetic changes occurring in the condition of organoid culture vs. parental/xenografts.

Based on the paper by Heide et al., concerns could emerge about how to consider our discordant classification of MI and NMI components. The variation of expression could be closely related to morphological, and mostly molecular, heterogeneity in bladder cancer. We might interpret this intratumor heterogeneity as an expression of the aforementioned cell plasticity. Nevertheless, this hypothesis cannot be assumed, and might be a consequence of an early clonal divergence that follows parallel developments occurring at different stages of disease progression [32]. 

Our findings could have relevant therapeutic implications, as tumors with a basal phenotype are most effective in MIBC to neoadjuvant CT than a luminal phenotype [33]. The basal phenotype in a superficial region (pTis/pTa) of the tumor could suggest a similar molecular feature, even in cystectomy, where the muscle invasive region of the tumor is thoroughly evaluated. Conversely, as we document in the papillary tumors group, a superficial luminal or mixed phenotype could change more commonly in the invasive UC component, implicating an unpredictable phenotype: a subset of patients could switch, acquiring basal markers, and resulting in a tumor more sensitive to CT.

Our results are also in line with the recent paper by Warrick JI et al. who, studying intratumoral molecular variability in bladder carcinoma, find that basal–squamous carcinomas often co-occur with carcinoma of another molecular subtype. The authors also hypothesize a sampling error in molecularly heterogeneous tumors, where the molecular subtyping from limited samples could misinform the CT response, and, therefore, treatment recommendations [34]. As of now, no immunohistochemical classification based on luminal and basal markers is available for clinical practice. The few data from the literature derive from small cohorts, and mostly from pTa/T1 tumors. Our proposed system, consisting of a semiquantitative score evaluation, demonstrates itself as a feasible, fast, and cost-effective method to use in daily practice. Moreover, it takes into account all major molecular phenotype-associated markers, and it could represent a screening tool able to assign patients to the main categories defined in the present molecular classification [16], possibly followed by NGS sub-classification. Using immunohistochemistry as the first step of the characterization has some advantages compared to NGS profiling. In particular, it allows an in situ tissue evaluation to distinguish the real tumor markers expression from a misleading stromal positivity (i.e. CD44 expression in the muscular wall of the bladder), thus, preventing potential overestimation of gene expression.

Our immunohistochemical results are supported by RT-PCR analysis of mRNA expression. For clinical purpose, knowing in advance the molecular phenotype of the tumor, as for the basal phenotype, could allow more accurate predicting, and at earlier stages (since pTa/pT1), of the probability of response to NAC. On the contrary, the molecular change toward a basal phenotype that we observe in luminal and mixed tumors from NMI to MI may have clinical implications, since their behavior and their sensitivity to therapies could be unpredictable during the early stages of disease. Furthermore, the complete loss of markers expression in the invasive component of a tumor (neu group) appears to be a negative prognostic factor, allowing the use of different treatments instead of cisplatin. Our study has some limitations: the retrospective nature of the analysis, and the lack of a standardized score system for immunohistochemical markers in the literature, which also prevents a better comparison between our results and other published studies. Although promising for the management of patients with urothelial carcinoma, our results should be validated and standardized with further prospective analyses. Furthermore, in TURB patients, the evaluation of immunophenotypical variability certainly requires suitable samples, including the muscularis propria (bladder resections often only have superficial sampling), and an en-bloc resection if feasible, for a better staging [35,36].

## 5. Conclusions

Our findings show an immunophenotypical classification score (Piescore), which works as a surrogate to discriminate between different molecular features of bladder cancer. Our scoring system reveals, for the first time, an intratumoral phenotypical change between NMI and MI bladder carcinoma. This molecular switch is common in mixed and luminal tumors, but not in basal tumors, which have a more stable phenotype. The molecular intratumoral variability that we observe could partially explain the sensitivity of a subset of luminal UC to CT: good responders could be “non-real” luminal UCs, which acquire basal markers, such as *CD44,* during progression. Prospective clinical studies remain mandatory to confirm our data, and translate them into clinical practice.

## Figures and Tables

**Table 1:** Demographic, pathologic data of patients and phenotypical stratification in MI component.

Variables		Luminal N (%)	Basal N (%)	Mixed N (%)	Neu-like N (%)
Patients (%)	251 (100)	73 (29.08)	107 (42.63)	21 (8.37)	50 (19.92)
Age					
Median	70.13	69.31	70.26	65.54	73.16
Gender					
Male	205 (81.67)	64 (31.22)	82 (40.00)	18 (8.80)	41 (20.00)
Female	46 (18.32)	9 (19.56)	25 (54.35)	3 (6.52)	9 (19.56)
Smoking					
Yes	121 (48.21)	44 (36.36)	45 (37.19)	11 (9.09)	21 (17.35)
Ex	26 (10.36)	6 (23.08)	17 (65.38)	0	3 (11.54)
No	58 (23.11)	12 (20.70)	26 (44.83)	7 (12.07)	13 (22.41)
n.a.	46 (18.33)	11 (23.91)	19 (41.30)	3 (6.52)	13 (28.26)
Tumor history (TURBT)					
Primary	219 (87.25)	60 (27.40)	97 (44.29)	20 (9.13)	42 (19.18)
Secondary	32 (12.75)	13 (40.62)	10 (31.25)	1 (3.12)	8 (25.00)
CIS (TURBT)					
Yes	38 (15.14)	15 (39.47)	14 (36.84)	4 (10.53)	5 (13.16)
No	213 (84.86)	58 (27.23)	93 (42.86)	17 (7.98)	45 (21.13)
CIS (cystectomy)					
Yes	5 (1.99)	2 (40.00)	1 (20.00)	0	2 (40.00)
No	246 (98.01)	71 (28.86)	106 (43.08)	21 (8.54)	48 (19.51)
pT (cystectomy)					
pT0	24 (9.56)	4 (16.67)	8 (33.33)	3 (12.5)	9 (37.5)
pTis/Ta	8 (3.19)	2 (25.00)	4 (50.00)	0	2 (25.00)
pT1	9 (3.58)	4 (44.44)	4 (44.44)	1 (11.11)	0
pT2	29 (11.55)	11 (37.93)	11 (37.93)	3 (10.34)	4 (13.79)
pT3	139 (55.38)	37 (26.62)	67 (48.20)	8 (5.75)	27 (19.42)
pT4	42 (16.73)	15 (35.71)	13 (30.95)	6 (14.28)	8 (19.05)
pN-M (cystectomy)					
pN0	104 (41.43)	26 (25.00)	50 (48.08)	11 (10.58)	17 (16.34)
pN1-3	58 (23.11)	22 (37.93)	19 (32.76)	5 (8.62)	12 (20.69)
pM1	5 (1.99)	2 (40.00)	2 (40.00)	0	1 (20.00)
LVI					
Yes	102 (40.64)	41 (40.20)	26 (25.49)	8 (7.84)	27 (26.48)
No	149 (59.36)	32 (21.48)	81 (54.36)	13 (8.72)	23 (15.43)
Necrosis					
Yes	96 (38.25)	24 (25.00)	49 (51.04)	6 (6.25)	17 (17.71)
No	155 (61.75)	49 (31.61)	58 (37.42)	15 (9.68)	33 (21.29)
CT					
Yes	63 (25.1)	18 (28.58)	23 (36.51)	9 (14.28)	13 (20.63)
No	188 (74.9)	55 (29.25)	84 (44.68)	12 (6.38)	37 (19.68)

**Table 2:** Stratification of different histological subtypes and markers expression in invasive component.

			Histological Category in pT2 UC at TUR and Markers					
	Architecture		Histological Subtype/Variant					
Phenotype	Papillary 139 (%)	Not Papillary 112 (%)	Classic 157	Divergent 71	Sarcomatoid 5	Nested 5	Micropap/Plasmacytoid 2/3	Neuroend 8
Basal Markers+								
CD44	60 (43.16)	51 (45.53)	55 (35.03)	49 (69.01)	4 (80)	2 (40)	0/0	1 (12.5)
CK 5/6	58 (41.73)	42 (37.5)	45 (28.67)	50 (69.44)	2 (40)	3 (60)	0/0	0
Luminal Markers+								
CK20	26 (18.70)	29 (25.89)	43 (27.39)	9 (12.5)	0	2 (40)	1 (50)/0	0
pPARg	27 (19.42)	35 (31.25)	43 (27.39)	13 (18.05)	0	3 (60)	2 (100)/0	1 (12.5)
Basal type 107 pts	63 (45.32)	44 (39.28)	51 (32.48)	49 (69.01)	5 (100)	2 (40)	0/0	0
Luminal type 73 pts	36 (25.90)	37 (33.03)	59 (37.58)	11 (15.28)	0	1 (20)	2 (100)/0	0
Mixed type 21 pts	12 (8.63)	9 (8.03)	15 (9.55)	4 (5.55)	0	1 (20)	0/0	1 (12.5)
Neu-like type 50 pts	28 (20.14)	22 (19.64)	32 (20.38)	7 (9.86)	0	1 (20)	0/3 (100)	7 (87.5)
Switched cases								
Yes 96 pts	64 (46.04)	32 (28.57)	63 (40.13)	23 (32.4)	3 (60)	2 (40)	0/3 (100)	2 (25)
No 155 pts	75 (53.96)	80 (71.43)	94 (59.87)	48 (67.6)	2 (40)	3 (60)	2 (100)/0	6 (75)
Pts 251 (100)	139 (100)	112 (100)	157 (100)	71 (100)	5 (100)	5 (100)	5 (100)	8 (100)

**Table 3:** Stratification of patients in the four phenotypes using the different marker combinations.

Phenotypes	Marker Combinations				Tot (%)
	CD44	CK5/6	CK20	pPARγ	
Basal (107)	+	+	-	-	74 (69.16)
	+	+	-	+	10 (9.3)
	+	+	+	-	2 (1.87)
	+	-	-	-	13 (12.15)
	-	+	-	-	8 (7.5)
Luminal (73)	-	-	+	+	30 (41.1)
	+	-	+	+	4 (5.5)
	-	+	+	+	3 (4.1)
	-	-	-	+	15 (20.5)
	-	-	+	-	21 (28.8)
Mixed (21)	+	+	+	+	4 (19.05)
	+	-	-	+	4 (19,05)
	-	+	+	-	0 (0)
	+	-	+	-	5 (23.8)
	-	+	-	+	8 (38.1)
Neu-like (50)	-	-	-	-	50 (100)

**Table 4:** Distribution of phenotypical transition in the different categories.

Cases Switch	*n*	*p* (*t* Test)
Yes No	96 155	-
Luminal vs. basal Yes No	53 vs. 8 68 vs. 63	<0.00001
Luminal vs. mixed Yes No	53 vs. 30 68 vs. 9	0.000318
Luminal vs. neu Yes No	53 vs. 5 68 vs. 15	ns
Basal vs. mixed Yes No	8 vs. 30 63 vs. 9	<0.00001
Basal vs. neu Yes No	8 vs. 5 63 vs. 15	ns
Mixed vs. neu Yes No	30 vs. 5 9 vs. 15	0.000121

## Data Availability

The data presented in this study are available on request from the corresponding author. The data are not publicly available, due to ethical considerations related to the privacy of medical data of patients included in this study.
